# Expanding the fragrance chemical space for virtual screening

**DOI:** 10.1186/1758-2946-6-27

**Published:** 2014-05-22

**Authors:** Lars Ruddigkeit, Mahendra Awale, Jean-Louis Reymond

**Affiliations:** 1 Department of Chemistry and Biochemistry, University of Bern, Freiestrasse 3, 3012 Bern, Switzerland

## Abstract

The properties of fragrance molecules in the public databases SuperScent and Flavornet were analyzed to define a “fragrance-like” (FL) property range (Heavy Atom Count ≤ 21, only C, H, O, S, (O + S) ≤ 3, Hydrogen Bond Donor ≤ 1) and the corresponding chemical space including FL molecules from PubChem (NIH repository of molecules), ChEMBL (bioactive molecules), ZINC (drug-like molecules), and GDB-13 (all possible organic molecules up to 13 atoms of C, N, O, S, Cl). The FL subsets of these databases were classified by MQN (Molecular Quantum Numbers, a set of 42 integer value descriptors of molecular structure) and formatted for fast MQN-similarity searching and interactive exploration of color-coded principal component maps in form of the FL-mapplet and FL-browser applications freely available at http://www.gdb.unibe.ch. MQN-similarity is shown to efficiently recover 15 different fragrance molecule families from the different FL subsets, demonstrating the relevance of the MQN-based tool to explore the fragrance chemical space.

## Background

Fragrance molecules are relatively small, lipophilic and volatile organic compounds that trigger the sense of smell by interacting with olfactory receptor neurons in the upper part of the nose which display a diverse array of olfactory G-protein coupled receptors [1-7]. These molecules are essential ingredient in foods, perfumes, soaps, shampoos or lotions, and can be classified according to their perceived smell into tens to hundreds of families [8]. Fragrance molecules form an important class of compounds, [9,10] and a sizable number of them have recently been collected in the public databases SuperScent [11] and Flavornet, [12] which list almost two thousand documented fragrance molecules and their properties.

However, global chemical space analyses of fragrance molecules have only been very limited so far [13,14]. Chemical space is understood as the ensemble of all organic molecules in the context of drug discovery, [15-27] and comprises millions of known molecules collected in public databases such as PubChem, [28] ChemSpider, [29] ZINC, [30]or ChEMBL, [31] and an even much larger number of theoretically possible molecules such as the Chemical Universe Databases GDB-11, [32,33] GDB-13 [34] and GDB-17, [35] listing all organic molecules possible up to 11, 13, and 17 atoms obeying simple rules for chemical stability and synthetic feasibility [30-33]. Herein we used the concept of chemical space to analyse and visualize fragrance molecules. Starting from the public databases Superscent and Flavornet, a “fragrance-like” property range was defined, and used to expand the fragrance chemical space by extracting fragrance-like molecules from the public databases ChEMBL, PubChem, ZINC and GDB-13 to form the corresponding fragrance-like subsets ChEMBL.FL, PubChem.FL, ZINC.FL and GDB-13.FL. The resulting fragrance-like chemical space was then analyzed using Molecular Quantum Numbers (MQN), a set of 42 simple integer value descriptors that count atoms, bonds, polar groups and topological features such as cycles. MQN provide a simple classification system for large databases with good performance in prospective virtual screening [36,37] as well as for database visualization [38,39]. The MQN-space approach was used to classify and represent the fragrance-like chemical space in form of an interactive application, the FL-mapplet, which is adapted from a previously reported MQN-mapplet application for the focused FL chemical space (freely available from http://www.gdb.unibe.ch) [40,41]. FL-molecules stand out from this visualization as being relatively simple due to the low number of heteroatoms and functional groups, and therefore appealing from the point of view of organic synthesis.

Fragrance chemistry is constantly searching for new fragrance molecules. A series of 15 different subsets of fragrance molecules were extracted from the SuperScent database and used to test ligand-based virtual screening (LBVS). MQN-similarity sorting enabled the efficient recovery of these known fragrance molecule families from the various FL subsets with equal or better performance that binary substructure fingerprints (Sfp) or extended connectivity fingerprints (ECfp4), illustrating the relevance of the MQN-classification with regards to fragrance molecule properties. The search for MQN-nearest neighbours is enabled by the FL-browser, which might serve as as a guide to identify new fragrance molecules.

## Results and discussion

### Property profiles

Molecules from the public databases SuperScent [11] and Flavornet [12] were assembled to form a collection of 1760 different fragrance molecules, here named FragranceDB. For comparison the databases BitterDB [42] listing 606 molecules with documented bitter taste and SuperSweet [43] listing 342 molecules with proven or likely sweet taste were combined to 806 taste molecules here named TasteDB, a diverse set of molecules whose diversity can be explained by the different types of receptors involved in recognition of sweet and bitter taste [44]. The molecular properties of FragranceDB and TasteDB was then analyzed in comparison to PubChem, [26] ChEMBL, [29] ZINC, [28] and GDB-13 [31] as representative databases of the broader chemical space (Table 1).

**Table 1:** Databases of molecules used in this work

**Database**	**Description**	**Size**	**Web addresses**
SuperScent	Database of scents from literature	1,591	http://bioinf-applied.charite.de/superscent/
Flavornet	Volatile compounds from literature based on GC-MS	738	http://flavornet.org
SuperSweet	Database of carbohydrates and artificial sweeteners	342	http://bioinf-applied.charite.de/sweet/index.php?site=home
BitterDB	Database of bitter Cpds from literature and Merck index	606	http://bitterdb.agri.huji.ac.il/bitterdb/
PubChem	NIH repository of molecules	48.8 M	http://pubchem.ncbi.nlm.nih.gov
ZINC	Commercial small molecules	13.5 M	http://zinc.docking.org
ChEMBL	Bioactive drug-like small molecules annotated with experimental data	1.5 M	https://www.ebi.ac.uk/chembldb
GDB-13	possible small molecules up to 13 atoms of C, N, O, S, Cl	980 M	http://gdb.unibe.ch
FragranceDB	SuperScent + Flavornet	1,760	http://gdb.unibe.ch
TasteDB	SuperSweet + BitterDB	806	http://gdb.unibe.ch
FragranceDB.FL	Fragrance-like subset of FragranceDB	1,475	http://gdb.unibe.ch
ChEMBL.FL	Fragrance-like subset of ChEMBL	10,373	http://gdb.unibe.ch
PubChem.FL	Fragrance-like subset of PubChem	566,870	http://gdb.unibe.ch
ZINC.FL	Fragrance-like subset of ZINC	37,662	http://gdb.unibe.ch
GDB-13.FL	Fragrance-like subset of GDB-13	59,482,898	http://gdb.unibe.ch

**Figure 1:**
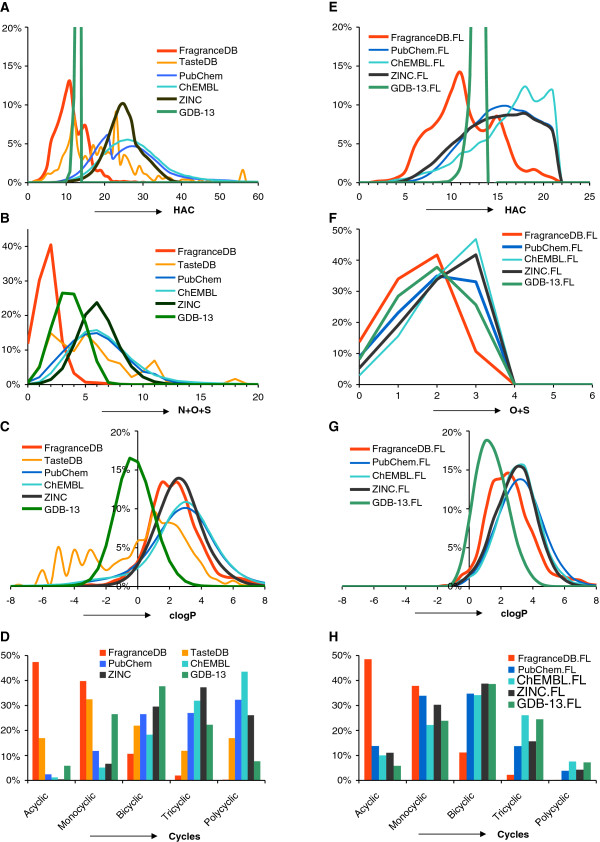
**Property histograms of various databases (A-D) and their fragrance-like subsets (E-H).** The frequency peak in FragranceDB at 9-11 heavy atoms corresponds to a diverse constellation comprising aliphatic linear and branched alkenes, aldehydes, alcohols, ketones and esters, various simple benzene, phenol and benzaldehyde analogs, furanones, monoterpenes. The frequency peaks in TasteDB at 10-12 atoms corresponds to various hexoses and their reduced hexitols, monoterpenes, coumarins, anisols, and amino acids.

The heavy-atom count (HAC) profile showed that FragranceDB comprised mostly fragment-sized [45] organic molecules with an upper boundary at approximately 21 atoms (Figure 1A). Most of the FragranceDB molecules were in the range of 5–17 heavy atoms. In contrast the molecules in PubChem, ChEMBL and ZINC peaked at the size of 20–30 heavy atoms, and TasteDB covered a broad size range. FragranceDB also stood out by a very low number of heteroatoms peaking at just two heteroatoms, mostly oxygens in volatiles aldehydes and ketones, alcohols, carboxylic esters and acids (Figure 1B). PubChem, ChEMBL and ZINC molecules contained more heteroatoms than FragranceDB molecules due to their larger size and high density of nitrogen-rich functional groups which are almost entirely absent in fragrance molecules. GDB-13 molecules also displayed more heteroatoms than FragranceDB molecules despite of their smaller size due to a combinatorial enumeration favoring highly functionalized molecules. The heteroatom profile of TasteDB was much broader, in line with the broader range of molecular weights, mostly as a consequence of the abundance of sweet tasting oligosaccharides including the steviol glycosides with a high density of hydroxyl groups [46].

In terms of polarity as estimated by the calculated octanol/water partition coefficient clogP, FragranceDB overlapped nicely with PubChem, ChEMBL and ZINC by covering the range 0 < clogP < 5, which is a polarity range suitable for rapid diffusion in biological media (Figure 1C). This probably reflects the necessity of fragrance molecules to diffuse from the gas phase to the olfactory neurons to reach their receptors, which requires properties similar to those necessary for drugs to reach their site of action. This property was also shared by the majority of TasteDB, however in this case a significant fraction of the database extended into negative clogP values, comprising mono-saccharides, disaccharides and related polyols, steviol glycosides, and amino acids and peptides such as aspartame. GDB-13, which reflects the combinatorial enumeration of the entire chemical space, peaked at clogP = 0 due to the large fraction of cationic polyamines in the database which extend into negative clogP values. Due to its size GDB-13 however still contained an extremely large number of molecules in the polarity range of fragrance molecules compared to the other databases.

FragranceDB further stood out as a collection of acyclic and structurally flexible molecules, with an abundance of acyclic aliphatic alcohols, aldehydes, acids and esters found for example in butter and fruit aroma (Figure 1D). Monocyclic molecules were also abundant, in particular cyclic terpenes such as limonene or menthol and aromatics such as cinnamaldehyde. By comparison PubChem, ChEMBL and ZINC were more abundant in polycyclic molecules due to the larger size of their molecules and the tendency to use rigid molecules for medicinal chemistry. On the other hand the combinatorial enumeration in GDB-13, which corresponds to the size-range of fragrance molecules, featured bicyclic molecules as the most frequent topology. TasteDB contained mostly monocyclic molecules, many of which were mono-saccharides, but also extended into polycyclic molecules due to the presence of oligosaccharides and steroids in the collection.

### Fragrance-likeness and fragrance-like subsets

The property profiles above indicated that fragrance molecules formed a family of relatively small molecules with a low number of heteroatoms and few cycles, in contrast to taste molecules in TasteDB and drug-like molecules which covered a much broader range of structural properties. A simple “fragrance-like” (FL) property range was defined as molecules with HAC ≤ 21 containing only carbon, hydrogen, oxygen or sulfur atoms, with a maximum of three heteroatoms (S + O ≤ 3) and maximum one hydrogen-bond donor atom (HBD ≤ 1). These FL criteria retained 84% of the molecules listed in the combined database (FragranceDB) and were used to define the fragrance like subsets PubChem.FL (1.2% of PubChem), ChEMBL.FL (0.68% of ChEMBL), ZINC.FL (0.28% of ZINC) and GDB-13.FL (6.1% of GDB-13) (Table 1). Note that excluding nitrogen containing molecules from FL criteria eliminated important fragrance molecules such as pyrazines, however the extremely large number of nitrogen containing molecules in the reference databases rendered any nitrogen-containing subsets too strongly enriched in this molecule class which forms only a minor fraction of fragrance molecules.

The property profiles of the FL-subsets showed that FL criteria brought the subsets within the range of FragranceDB. In the HAC profile however, PubChem.FL, ChEMBL.FL and ZINC.FL peaked in the range 15–21 atoms following the abundance of larger molecules in the parent databases, which is substantially higher than the abundance peak of FragranceDB. GDB-13.FL had a sharp abundance peak at HAC = 13 like its parent database GDB-13 (Figure 1E). Most FL molecules from these databases contained three heteroatoms (S + O) while FragranceDB peaked at only two heteroatoms (Figure 1F). Nevertheless FL molecules from PubChem.FL, ChEMBL.FL and ZINC.FL had a somewhat higher clogP indicating higher lipophilicity reflecting their somewhat larger size at similar number of heteroatoms (Figure 1G). GDB-13.FL had a lower clogP value distribution due to the combinatorial enumeration of heteroatom substitutions giving a larger number of possibilities at high numbers of heteroatoms. In contrast to FragranceDB which contains mostly acyclic molecules, the FL subsets were most abundant in monocyclic and bicyclic molecules, again reflecting either the larger molecular size in PubChem.FL, ChEMBL.FL and ZINC.FL, or the larger diversity of cyclic structures formed by combinatorial enumeration in GDB-13.FL (Figure 1H).

### Interactive visualization of the fragrance chemical space

**Figure 2:**
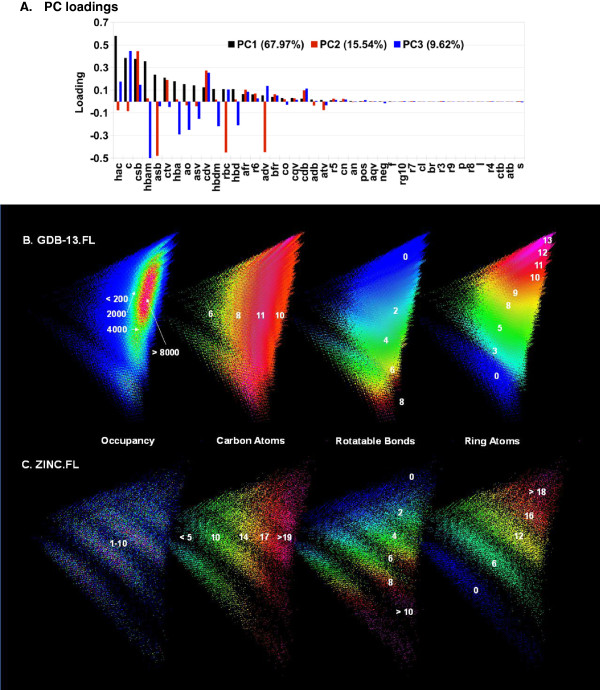
**Color-coded MQN-maps of subsets GDB-13-FL and ZINC.FL. A**. Loadings of PC1, PC2 and PC3 for PCA of FragranceDB. The 42 MQNs are defined as follows: atom counts: c, f, cl, br, i, s, p = elements, an/cn = acyclic/cyclic nitrogens, ao/co = acyclic/cyclic oxygens, hac = heavy atom count, bond counts: asb/adb/atb = acyclic single/double/triple bonds, csb/cdb/ctb = cyclic single/double/triple bonds, rbc = rotatable bond count, polarity counts: hba/hbd/hbam/hbdm = H-bond acceptor/donor atoms/sites, pos/neg = positive/negative charges at pH 7.4, topology counts: asv/adv/atv/aqv = acyclic monovalent/divalent/trivalent/tetravalent nodes, cdv/ctv/cqv = cyclic divalent/trivalent/tetravalent nodes, r*i* = *i*-membered rings (*i* = 3-9), rg10 = ≥10-membered rings, afr/bfr = atoms/bonds shared by fused rings. r*i*, rg10 and afr/bfr are counted in the smallest set of smallest rings.**B**. Color-coded maps for GDB-13.FL. Note that the carbon count decreases at right because heteroatom rich compounds take over. **C**. Color-coded maps for ZINC.FL. Color-coding represents the increasing value of the indicated property in the scale blue-cyan-green-yellow-orange-red-magenta. Interactive color-coded MQN-maps for all FL subsets can be accessed with the FL-mapplet at http://gdb.unibe.ch.

Visualization and understanding of implicit features of high-dimensional property spaces often require use of dimensionality reduction techniques, which project the data on a 2D plane, while keeping most of geometric information from the original space. One such technique is a Principal Component Analysis (PCA), which we have used in previous studies for visualization of large databases [40]. Here, FragranceDB and the corresponding FL subsets of larger databases defined above were analyzed by MQN for visualisation. In the PCA of FragranceDB, PC1 covered 67.97% of the variance with positive loadings in all descriptors, corresponding to molecular size (Figure 2A). PC2 covered 15.54% of the variance with negative loadings for counts of acyclic atoms and bonds and positive loadings for descriptors of cyclic atoms and bonds. PC3 accounted for a further 9.62% of variance representing polarity descriptors such as H-bond donor atoms. The loadings were similar for the other FL subsets.

To provide a uniform visualization all FL subsets were represented in the (PC1, PC2)-plane corresponding to the PCA of FragranceDB. As illustrated for GDB-13.FL (Figure 2B) and ZINC.FL (Figure 2C), the layout was similar to that observed previously with MQN datasets of a variety of small molecule databases [40]. The MQN-maps appeared as a left-point triangle containing parallel diagonal stripes corresponding to groups of molecules with an increasing number of cycles. In these maps small molecules appeared at left and large molecules at right, acyclic molecules at bottom and polycyclic molecules at the top. Due to the heteroatom restrictions imposed in the FL criteria, the depth of the FL subsets in the PC3 dimension spanning polarity was rather limited.

An interactive FL-mapplet was then generated by modifying the data in the previously reported MQN-mapplet application [40]. This Java application allows to directly view the structural formulae of compounds in each pixel of color-coded MQN-maps, and to subsequently access the compound information at the source database (e.g. DrugBank, ChEMBL, ZINC, PubChem). The FL-mapplet was also linked to the MQN-browser for fragrance molecules to enable MQN-nearest neighbour searches (see below). Similarly to the MQN-mapplet, the FL-mapplet can be downloaded as a Java application from gdb.unibe.ch, and contains a link to the same help page providing detailed explanations on how to use the application.

The main advantage of the interactive FL-mapplet is that one can rapidly inspect the structural formulae of the molecules in the various FL-subsets prearranged in the logical layout of the MQN based PCA maps. One of the striking aspects seen by inspecting the FL subsets is that FL-molecules are relatively simple due to the low number of heteroatoms and functional groups. FL compounds are clearly appealing from the point of view of organic synthesis because of their low number of polar functional groups which draws attention to the carbon skeletons classically at the center of synthesis planning. Concerning the FL-subsets presented here, inspecting GDB-13.FL where almost all molecules are novel might prove particularly inspiring for designing new yet tractable synthetic targets in the fragrance chemical space [47,48].

### Ligand-based virtual screening in the FL chemical space

Although fragrance molecules interact simultaneously with hundreds of different olfactory receptors, structure-activity relationships (SAR) in these compounds are not fundamentally different from those of drug-receptor interactions [13,14]. Certain compound classes are well correlated with fragrance types, e.g. short chain aliphatic esters with fruity flavors. On the other hand completely different compound classes may elicit the same smell, for example the very different types of musks. Furthermore subtle differences such as chirality may erase the fragrant property or completely switch the fragrance type, e.g. the classical case of (−)- and (+)-carvone displaying spearmint respectively caraway flavor [49]. Despite of many such cases of extreme sensitivity of activity to structural alterations representing activity cliffs in the SAR landscape, [50] we asked the question whether ligand-based virtual screening (LBVS) in the FL subsets, as is used to identify drug analogs, might also by useful to identify fragrance molecule analogs. To the best of our knowledge a systematic study of LBVS in the fragrance chemical space is unprecedented [51,52].

To test this hypothesis, fragrance molecule families were retrieved from the Superscent tree with the condition that they contained at least 10 molecules after removal of molecules listed in more than five different families and those not following FL criteria, which eliminated promiscuous compounds such as dimethyl disulphide, cyclopentanethiol or 3-ethyl pyridine, and nitrogen containing compounds such as ethyl antranilate or pyrazine. This procedure gave 15 sets of fragrance molecules containing between 10 and 122 compounds each, consisting mostly of alcohols, aldehydes and esters (Table 2 and Additional files 1, 2 and 3). LBVS by MQN-similarity was performed for FragranceDB and the various FL subsets and compared with recovery using a Daylight-type 1024 bit substructure fingerprint (Sfp), [53] the extended connectivity fingerprint ECfp4, [54] and the molecular weight (MW). The city-block distance (CBD) was used for all similarity calculations since CBD performs as well as the Tanimoto similarity but is much easier to compute, enables rapid browsing (see below), and directly relates to the concept of chemical space [39,41]. For each fingerprint, the compound closest to all other compounds in the family was chosen as reference compound, and the receiver operator characteristic (ROC) curve was calculated.

**Table 2:** Recovery of fragrance molecule families from various databases

***Fragrance***	***Cpds nr.***	***HAC av.***	***FragranceDB*** ***recov. at 10*%**	***PubChem.FL*** ***recov. at 1*%**	***ChEMBL.FL*** ***recov. at 1*%**	***ZINC.FL*** ***recov. at 1*%**	***GDB-13.FL*** ***recov. at 0.1*%**
Vegetable	10	7.20	**45**/0/22/**45**	**56**/0/44/11	**45**/0/11/0	**33**/0/22/0	**78**/22/67/56
Fishy	11	8.64	**40**/20/**40**/0	**40**/30/**40**/0	**50**/20/20/0	10/20/**40**/0	67/44/**78**/33
Chemical	23	8.87	**14**/**14**/9/9	14/**18**/9/0	5/5/**9**/0	5/**9/9**/0	37/37/**63**/21
Ethereal	14	8.93	**46/46**/23/8	36/**62**/23/8	46/**54**/15/8	23/**46**/15/8	55/**82**/55/45
Medicinal	12	9.58	55/**64**/55/9	55/**64**/55/9	**55**/46/37/9	**55/55**/36/9	67/**89/89**/56
Nutty	28	10.14	**37**/30/4/15	33/**37**/4/4	**22**/19/9/4	**19/19**/4/4	42/**54**/13/21
Fatty	42	10.36	17/**22**/15/12	10/**27**/20/7	**17/17**/5/7	7/**22**/5/2	33/45/**48**/3
Smoky	12	11.42	18/18/**36**/9	18/18/**27**/8	9/9/**18**/0	9/9/**18**/0	-
Fruity	122	11.56	**23/23**/5/16	17/**33**/8/2	19/**22**/1/8	11/**21**/2/2	35/**49**/36/0
Minty	13	11.92	**58**/8/50/33	**42**/0/**42**/8	**42**/0/34/8	**42**/0/**42**/8	**44**/0/22/22
Citrus	35	12.06	**29**/15/12/18	9/**18/18**/0	**36**/15/12/0	9/15/**18**/0	9/30/**43**/13
Balsamic	64	12.25	**30**/6/5/13	**19**/6/8/2	**14**/2/2/2	**5/5**/0/2	**39**/10/29/0
Floral	69	12.81	**22**/0/16/21	7/0/**12**/6	**9**/0/6/6	**6**/0/**6/6**	18/0/**43**/7
Herbaceous	13	12.92	**33**/17/8/17	**8**/0/0/**8**	**8**/0/0/**8**	**8**/0/0/**8**	-
Waxy	11	14.18	**60**/40/40/30	30/40/**90**/10	**50**/40/40/10	30/40/**70**/10	-
Average	32	10.86	**35**/22/23/17	26/24/**27**/5	**29**/17/14/5	18/17/**19**/4	44/39/**49**/23
No. of best scores per series			**12**/5/2/1	5/6/**6**/1	**11**/3/2/1	**7/7/7**/2	3/4/**6**/0

For each database the % actives found is given for the indicated % database screened by sorting with MQN/Sfp/ECfp4/MW similarity to the most average molecule in the set. The highest value in each entry is highlighted in bold. Fragrance families were collected from the Superscent database website. Compounds appearing in more than 5 different families and those not following FL criteria were removed. Data was not computed for GDB-13.FL if the families were smaller than 10 compounds after removal of HAC > 13 compounds. The city-block distance was used as similarity measure (results were comparable using Tanimoto).

**Figure 3:**
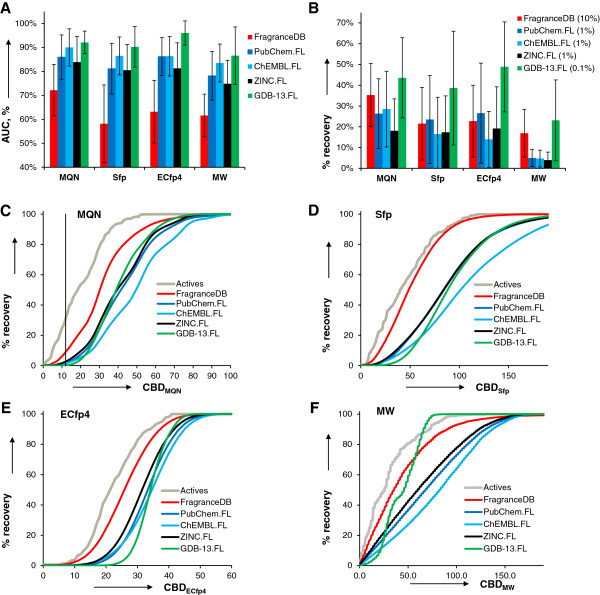
**LBVS of fragrance molecule analogs (15 sets from Table**2**). A**. Average AUC ± σ for recovery of the 15 fragrance molecule sets from the corresponding reference using MQN, Sfp, ECfp4 or MW. **B**. Average percentage of actives recovered ± σ at the indicated database coverage. **C-F**. Average cumulative recovery of actives and average coverage of each database as a function city-block distance from the reference compound of each active set, in MQN-space (CBD_MQN_, **C**), Sfp-space (CBD_Sfp_, **D**), ECfp4-space (CBD_ECfp4_, **E**), and MW-space (CBD_MW_, **F**). ROC-curves for each fragrance molecule family are available in the Additional file 1.

MQN, Sfp, ECfp4 and MW gave comparable performance in terms of the area under the curve (AUC), which was only slightly above the random selection value (AUC = 50%) for the very small FragranceDB collection but generally above 80% in the larger databases, indicating in particular that MW was a defining parameter in the selected fragrance molecule series (Figure 3A). Analysis of the recovery of actives as a function of the percentage of database screened however showed that MQN, Sfp and ECfp4 were much better at recovering the fragrance molecule series compared to MW in the early phase of recovery, which is most decisive in an LBVS application (Table 2, Figure 3B). This was the case at 10% screening of FragranceDB (corresponding to 148 nearest neighbours of each reference compound), 1% screening of PubChem.FL (5669 nearest neighbours), ChEMBL.FL (104 nearest neighbours) or ZINC.FL (377 nearest neighbours), and 0.1% screening of GDB-13.FL (595,000 nearest neighbours). MQN gave the highest recovery from FragranceDB in 12 of the 15 series, with an average of 35% recovery at 10% database screening. MQN also surpassed the other fingerprints in 11 series for recovery from ChEMBL.FL, with an average of 29% recovery at 1% database screening, and performed comparably well to ECfp4 and Sfp in PubChem.FL and ZINC.FL with an average of 26% and 18% recovery at 1% screening respectively. In the case of GDB-13.FL ECfp4 (average 49% recovery at 0.1% screening) was slightly better than MQN (average 44% recovery at 0.1% screening), while Sfp was somewhat less efficient (average 39% recovery at 0.1% screening).

The performance of LBVS for fragrance molecule analogs was further illustrated by displaying the average recovery of actives and of the various databases from the corresponding references as a function of the city-block distance (Figure 3C-F). MQN stood out from the other fingerprints by its ability to differentiate fragrance molecule analogs at low CBD over the other databases including FragranceDB. The sigmoidal shape of the recovery curve for MQN, Sfp and ECfp4, which was absent in the case of MW, illustrates why these fingerprints provide high enrichment factors of actives at low percentage coverage of the various databases.

Overall MQN performed as well as and sometimes better than ECfp4 and Sfp in LBVS for fragrance molecules despite the fact that Sfp and ECfp4 contain much more detailed representations of the molecular structure than MQN, suggesting that the MQN-based analysis and visualization presented above were relevant in terms of fragrance molecule properties. This observation confirmed our previous reports that MQN-similarity preforms quite well in LBVS of drug analogs such as the recovery of actives from decoys in the directory of useful decoys (DUD), [39,55] and the recovery of shape and pharmacophore analogs from GDB-13 [36,56].

### The FL-browser

Nearest neighbour searching by city-block distance in MQN-space can be carried out extremely fast even in extremely large databases when these are pre-organized by the sum of all MQN-values as hash-function [57]. A series of web-based MQN-browser applications are freely accessible at http://www.gdb.unibe.ch to perform such searches in various public databases by MQN-similarity [58]. To complement these applications the various FL subsets were formatted for CBD_MQN_ searches in a common web-based tool. In the resulting FL-browser, one can search in one or several of the various FL subsets simultaneously. As an example of MQN-similarity searching, we searched the MQN-space of ZINC.FL as a source of commercially available analogs, and of GDB-13.FL as a source of new compounds. The search was also carried out in the parent databases ZINC and GDB-13 using the corresponding MQN-browsers. Nearest neighbours searches were performed for 13 different classical fragrance molecules falling in the size-range of GDB-13, which are mostly monoterpenes (Table 3 and Additional file 4). The distance boundary CBD_MQN_ ≤ 12 was used because it was found to narrow the search to useful bioactive analogs in previous virtual screening studies [57]. A further limitation to isomers within the preset CBD_MQN_ distance boundary was also considered because isomerism further constrains the functional group and molecular size similarity, which are very important parameters in fragrance molecule properties. The MQN-browser for fragrance molecules offers options to search for isomers as well as to keep the number of H-bond donor atoms and H-bond acceptor atoms constant, which helps narrowing the search.

**Table 3:** **Number of fragrance molecule analogs found by nearest-neighbour searches in the MQN-space of ZINC, ZINC.FL, GDB-13 and GDB-13.FL within the distance boundary CBD**_**MQN**_ **≤ 12**

**Fragrance molecule**	**Formula**	**ZINC**	**ZINC.FL**	**Isomers**	**GDB-13**	**GDB-13.FL**	**Isomers**
Furaneol	C_6_H_8_O_3_	200	66	3	14412	2108	41
Isoamyl acetate	C_7_H_14_O_2_	3025	1332	38	164151	64056	540
Caprylic acid	C_8_H_16_O_2_	1437	735	14	427990	130781	28
Vanillin	C_8_H_8_O_3_	4771	614	18	397263	42394	899
Cinnamaldehyde	C_9_H_8_O	1403	446	13	26249	9160	223
Limonene	C_10_H_16_	773	323	18	112817	68672	2074
α-Pinene	C_10_H_16_	64	54	9	65614	158131	1549
Camphor	C_10_H_16_O	200	116	11	243162	158131	8397
Menthone	C_10_H_18_O	1147	424	43	605667	269391	5566
Rose oxide	C_10_H_18_O	889	402	44	624293	89209	7774
Menthol	C_10_H_20_O	734	282	26	383641	189579	1460
Citronellol	C_10_H_20_O	1642	621	38	2927465	910666	4674
Lauraldehyde	C_12_H_24_O	260	169	4	93700	50993	4748

**Figure 4:**
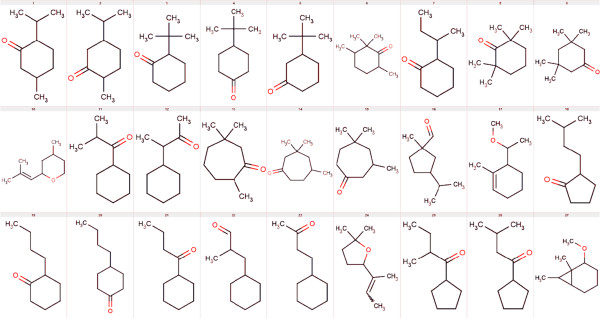
MQN-nearest neighbour isomers of menthone (hit no. 1) in the ZINC database preserving the same number of H-bond donor atoms (0) and H-bond acceptor atoms (1).

The MQN-neighbours of the peppermint fragrance component menthone are shown as an example (Figure 4). From the 424 commercially available compounds in ZINC.FL within CBD_MQN_ ≤ 12, we used the browser option to lock the number of H-bond donor atoms (0) and H-bond acceptor atoms (1) to restrict this selection further to 262 compounds, 27 of which were isomers of menthone. These analogs contained menthone itself (hit no. 1), a regioisomer (hit no. 2), but also various other cyclohexanones with the same number of acyclic carbon atom substituents (hits no. 3 to 9). Cycloheptanones (hit no. 13 – 15) and cyclopentanones (hit no. 26–27) were also proposed by the MQN-similarity search. When a similar search was carried out with GDB-13.FL, 4589 of the 5556 isomers had preserved H-bond donor and H-bond acceptor atom counts. The structural types encountered corresponded to those seen in ZINC but with exhaustive regiochemical enumeration and the addition of other scaffolds such as cyclobutanones and various cyclopropane containing scaffolds, most of which are not available in public domain as having physical samples.

## Conclusion

The general properties of fragrance molecules, which are relatively small organic compounds with few polar functional group such as to be volatile, were used to define a “fragrance-like” subset of the chemical space which was extracted from the public databases PubChem, ChEMBL, ZINC and GDB-13. The FL chemical space contains fragment-size, relatively non-polar molecules, and is clearly separate from the well-known drug-like chemical space [59]. The representation of the FL chemical space using interactive color-coded MQN- maps illustrates the extent of the structural diversity at hand. The corresponding FL-mapplet for interactive visualization (Java application to download) and FL-browser for fast MQN-similarity searching of the various FL subsets are freely accessible at gdb.unibe.ch. Inspecting fragrance molecules through these interactive tools shows that FL-molecules appear as particularly appealing from the point of view of organic synthesis due to the low number of heteroatoms and functional groups.

The fragrance chemical space, although relatively narrowly defined, is currently only relatively sparsely populated compared to its potential, implying that many millions of additional fragrance molecules remain to be discovered. Here we showed the MQN-similarity searching efficiently recovers known fragrance molecule families collected from SuperScent from the various FL subsets, with equal or better performance than substructure fingerprints Sfp of the extended connectivity fingerprint ECfp4. The ability to perform efficient LBVS by MQN-proximity searching as enabled by the FL-browser suggests that this resource might facilitate the identification of new fragrance molecules by rapidly pointing to compound series to be evaluated.

## Methods

### FragranceDB and TasteDB

Structure representations from SuperScent [11] were retrieved from their chemical classes’ folder. The list was inspected visually and in some few cases corrected. Names from Flavornet [12] were retrieved and converted by Molconvert from ChemAxon Pvt. Ltd (http://www.chemaxon.com/). Furthermore, in some cases Msketch (from ChemAxon) was used. Both datasets were combined and checked for duplicates to a final list of 1760 fragrance molecule structures. For TasteDB structure representations were retrieved from the browsing option of BitterDB [42] and from the Sweet-tree of SuperSweet [43]. Both datasets were combined and checked for duplicates to a final list of 806 taste structures.

### FL-mapplet and MQN-browser for fragrance molecules

The FL-mapplet has been adapted from our previously published MQN-mapplet [40] by mapping the various FL-subsets (Table 1) on the (PC1,PC2)-plane of the PCA calculated for FragranceDB (see Figure 2), creating the corresponding color-coded maps, and importing the data into the MQN-mapplet. For the PCA maps and assembly of FL-mapplet, PC1-PC2 plane was represented by 1000x1000 grid points (pixels), followed by the assignment of the each of the database molecule on to the grid. Each of the point (pixel) was colour coded according to the average and standard deviation of property (for e.g. heavy atom count) of molecules residing in that pixel. HSL colour space was used for the colour coding. Base colour (H) changes from blue-cyan-green-yellow-red-magenta with increasing average value of property in the pixel, while base colour fades towards the grey with increasing standard deviation. The average molecule for each of the pixel was the determined as follows: a) 42 average MQN values were determined considering MQNs of all of the molecules in given pixel b) City block distance was calculated between 42 MQN values of each of the molecule in the pixel and the 42 average MQN values c) molecule with lowest city block distance to average MQN values was considered as “average molecule” for the pixel.

FL-mapplet is a Java application. Details of the application usage are available on the help page accessible from within the application.

The MQN-browser for fragrance molecules is a web-based application which is accessible from within the FL-mapplet or directly at gdb.unibe.ch. This browser was programmed as previously described for the MQN-browser for other databases to allow nearest neighbour searching of any query molecules within the FL-subsets using CBD_MQN_ as similarity measure [57]. Searching in database space is enabled by use of bit mask values to store the database information of the structures. Bits were assigned to each database. During similarity searching, choice of databases made by user defined as “wanted bit mask” using Bitwise OR operation.

### Ligand-based virtual screening

Enrichment studies for the recovery of various fragrance molecule classes (actives) from the fragrance like databases (decoys) ChEMBL.FL, FragranceDB, PubChem.FL, ZINC.FL and GDB-13.FL were carried out using a java program written in-house using the JChem chemistry library from ChemAxon Ltd. as starting point. Fragrance classes were collected from the SuperScent database (http://bioinf-applied.charite.de/superscent/). Later, molecules within each of the fragrance class were filtered for duplicates and FL criteria. After processing, 15 fragrance classes containing at least 10 molecules in each, were retain for further study. In case of enrichment against GDB-13.FL, fragrance classes were additionally filtered to contain molecules with maximum of 13 heavy atoms. This results in the 12 fragrance classes with at least of 10 molecules in each of them.

Following the ionization of molecules at pH 7.4, Molecular Quantum Numbers (MQN, 42 dimensions), Daylight type binary substructure fingerprint (Sfp, 1024 bits, path length 7), circular Extended Connectivity fingerprint with bond diameter of 4 (ECfp4, 1024 bits) and Molecular weight (MW) were calculated for fragrance molecule classes and database molecules. Computation of molecular properties and fingerprints were enabled by JChem 5.4.1 Chemistry library from ChemAxon Pvt. Ltd. City block distance (CBD) was used as scoring function for virtual screening. Within each of the fingerprint space, enrichment studies were carried as follows: a) for each of the 15 fragrance molecule classes (defined above, 12 in case of GDB-13.FL) reference/query molecule was defined as compound which is most similar to all the other compounds (molecule with lowest CBD to all the other compounds) in the given fragrance molecule class. b) Each of the 15 fragrance molecule classes (12 in case of GDB-13.FL) was separately diluted in five FL like databases ((4*15) + 12 = 72 databases) c) diluted databases were screened against respective query molecule using city block distance as scoring function d) each of the screened database was sorted with increasing CBD to the query molecule, which was followed by the computation of ROC (receiver operator characteristic) curve, EF at 0.1%, 1% and 10%. Data in Figure 3A was obtained by averaging AUC values for 15 fragrance classes (12 in case of GDB-13.FL) within each of the fingerprint space.

## Competing interests

The authors declare that they have no competing interests.

## Authors’ contributions

LR designed and performed the study, database assembly and analysis, FL-browser and FL-mapplet, and wrote the paper. MA helped in the FL-mapplet assembly and performed the LBVS study, and wrote the paper. JLR designed and supervised the study and wrote the paper. All authors read and approved the final manuscript.

## Authors’ information

Web: http://www.gdb.unibe.ch.

## Supplementary Material

Additional file 1 **SMILES of fragrance molecules in each of the family in Table** 2**.** Click here for file

Additional file 2 **SMILES of the reference molecules used for LBVS examples in Table** 2**.** Click here for file

Additional file 3 **ROC curves for the LBVS examples in Table** 2**.** Click here for file

Additional file 4 **SMILES for the MQN-browser search examples in Table** 3**.** Click here for file
