# Supplementary material for: Expanding the fragrance chemical space for virtual screening
Source: J Cheminform. 2014 May 22;6:27. doi: 10.1186/1758-2946-6-27 (PMC4037718; doi:10.1186/1758-2946-6-27)
Supplement: Additional file 3 — ROC curves for the LBVS examples in Table 2. [file 1758-2946-6-27-S3.pdf]

Supporting Information for

Expanding the Fragrance Chemical Space for Virtual Screening

Lars Ruddigkeit, Mahendra Awale and Jean-Louis Reymond*

Department of Chemistry and Biochemistry, University of Bern, Freiestrasse 3, 3012 Bern, Switzerland, jean-louis.reymond@dcb.unibe.ch, web: gdb.unibe.ch

Table of Contents

ROC curves for Ligand-based virtual screening of fragrances in FragranceDB and the fragrance-like

subsets PubChem.FL, ChEMBL.FL, ZINC.FL, and GDB-13.FL...S2-S6

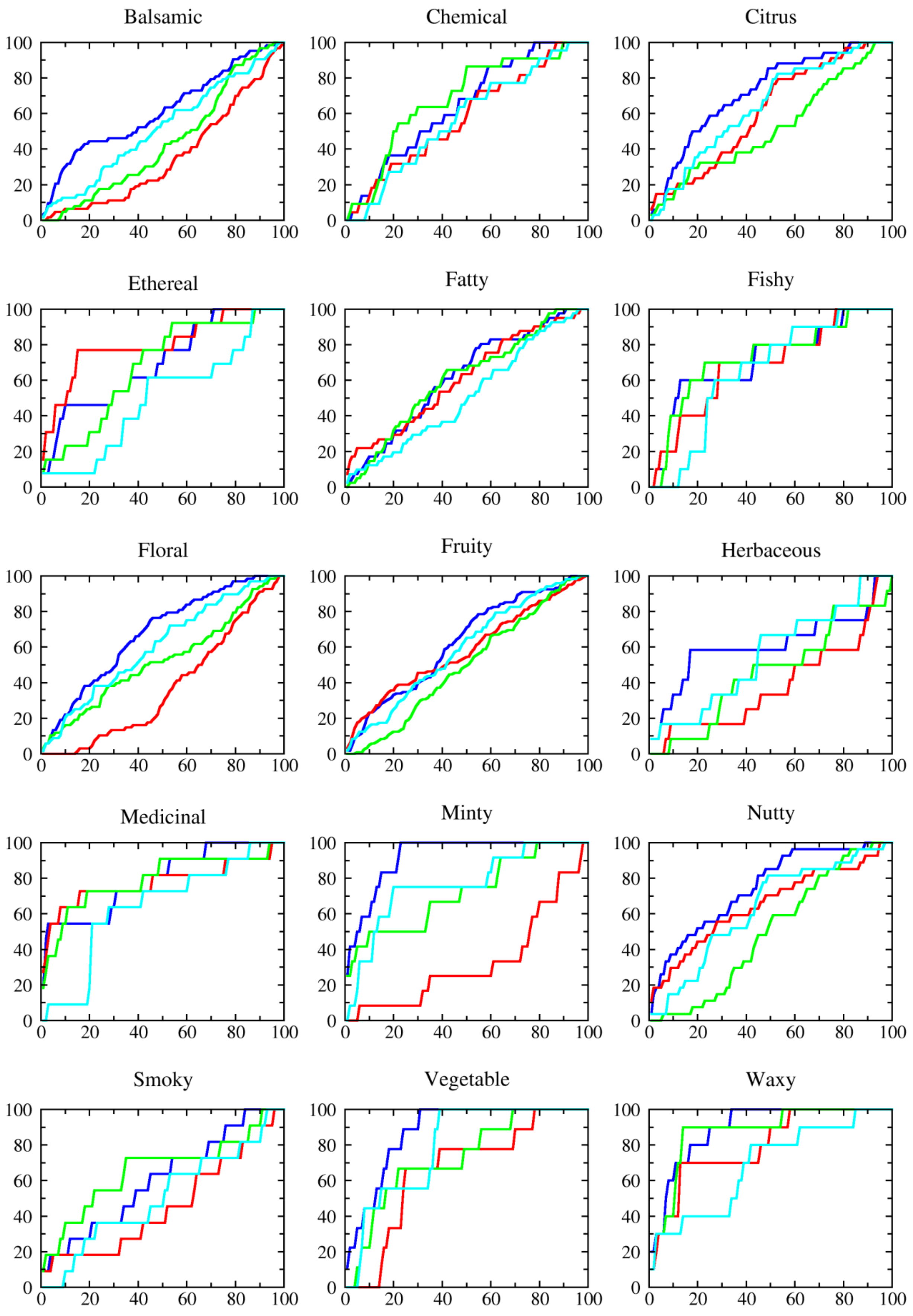

Figure S1. Receiver Operating Characteristic (ROC) curves for recovery of various fragrance classes against FragranceDB**,** using CBD_{MQN} (blue), CBD_{Sfp} (red), CBD_{ECfp4} (green), $CBD_{MolWt.}$(cyan). X-axis is % of sorted database and Y-axis is % of fragrances found.

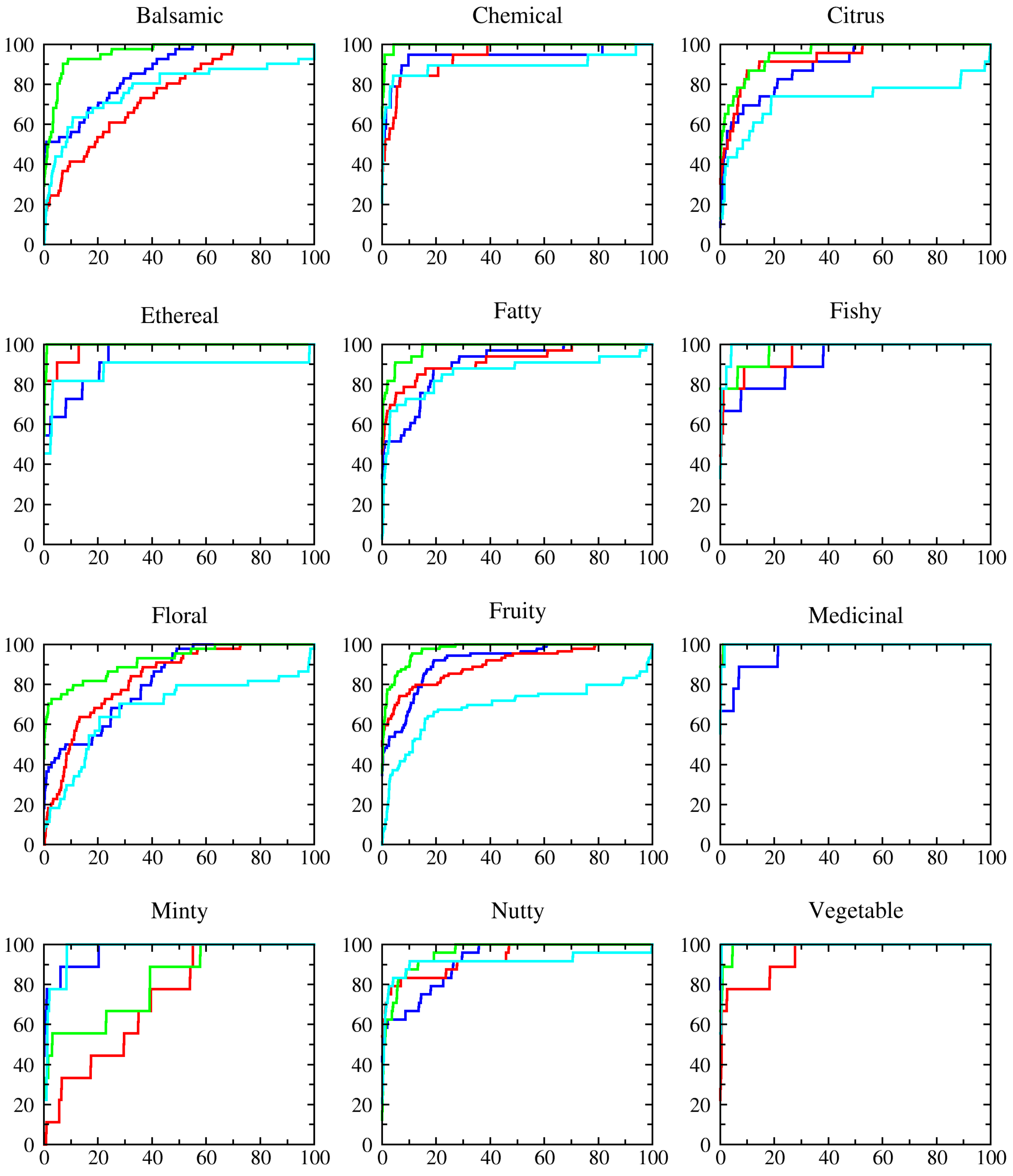

Figure S2. Receiver Operating Characteristic (ROC) curves for recovery of various fragrance classes against PubChem.FL**,** using CBD_{MQN} (blue), CBD_{Sfp} (red), CBD_{ECfp4} (green), $CBD_{MolWt.}$(cyan). X-axis is % of sorted database and Y-axis is % of fragrances found.

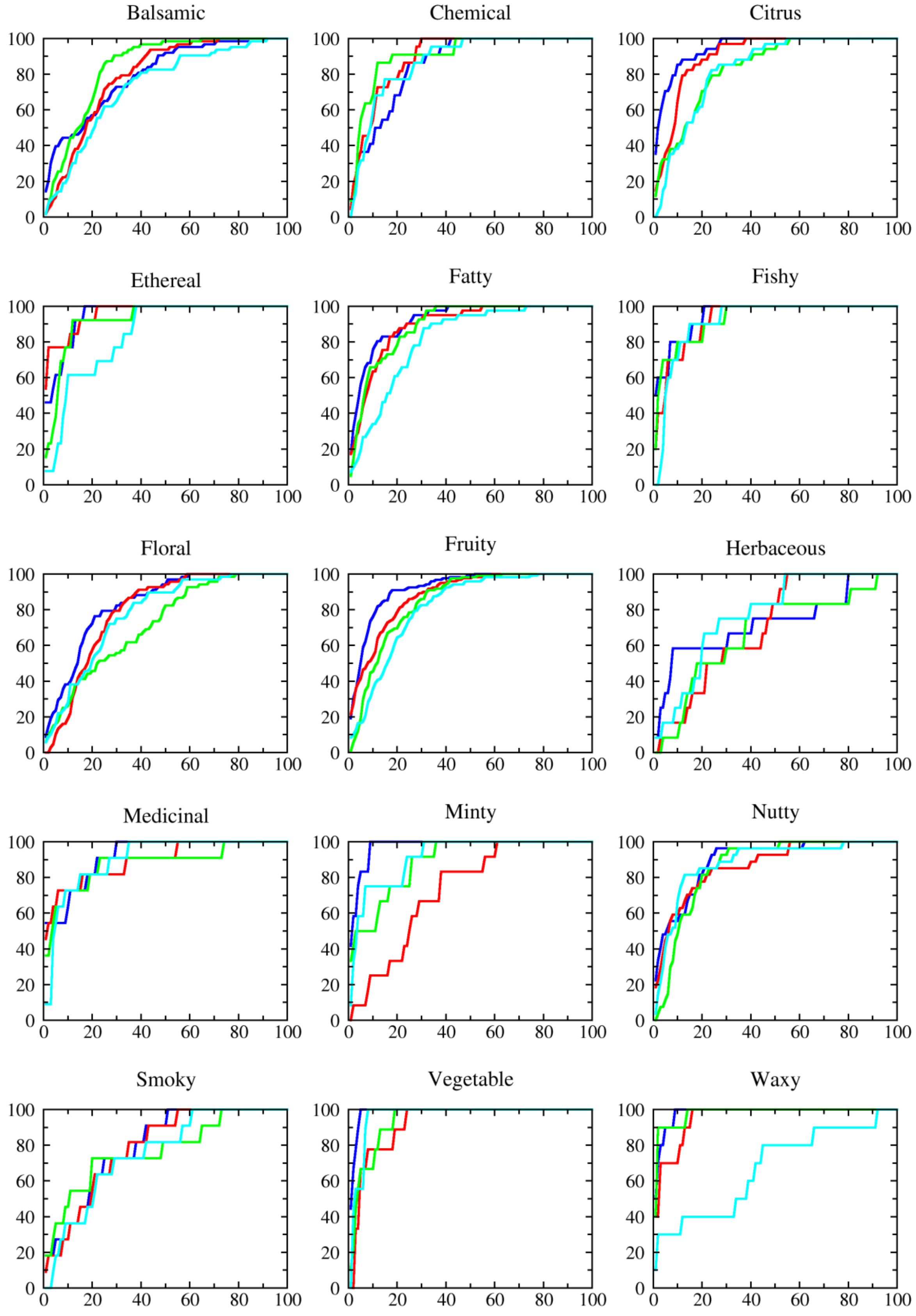

Figure S3. Receiver Operating Characteristic (ROC) curves for recovery of various fragrance classes against ChEMBL.FL database, using CBD_{MQN} (blue), CBD_{Sfp} (red), CBD_{ECfp4} (green), $CBD_{MolWt.}$(cyan). X-axis is % of sorted database and Y-axis is % of fragrances found.

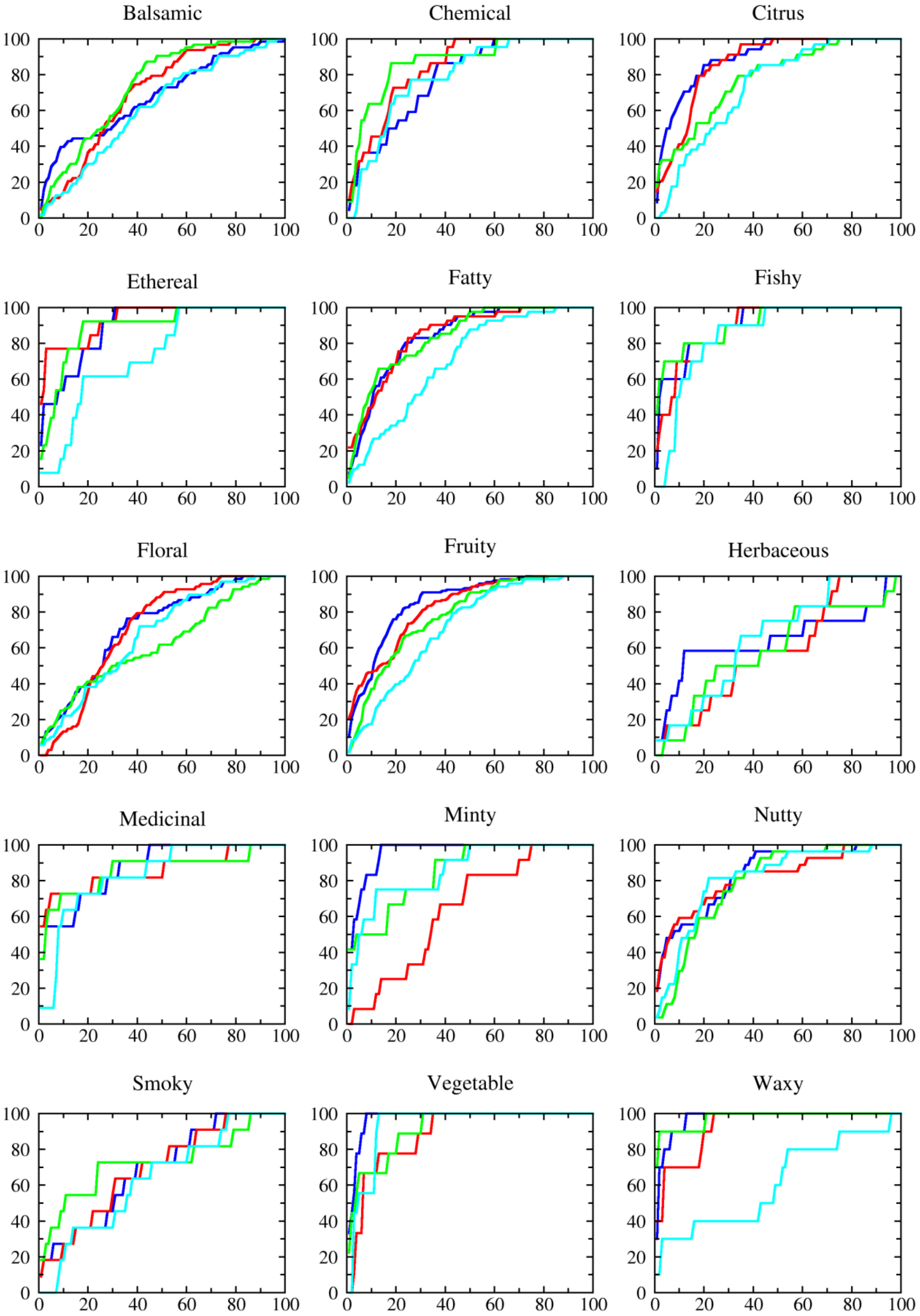

Figure S4. Receiver Operating Characteristic (ROC) curves for recovery of various fragrance classes against ZINC.FL**,** using CBD_{MQN} (blue), CBD_{Sfp} (red), CBD_{ECfp4} (green), $CBD_{MolWt.}$(cyan). X-axis is % of sorted database and Y-axis is % of fragrances found.

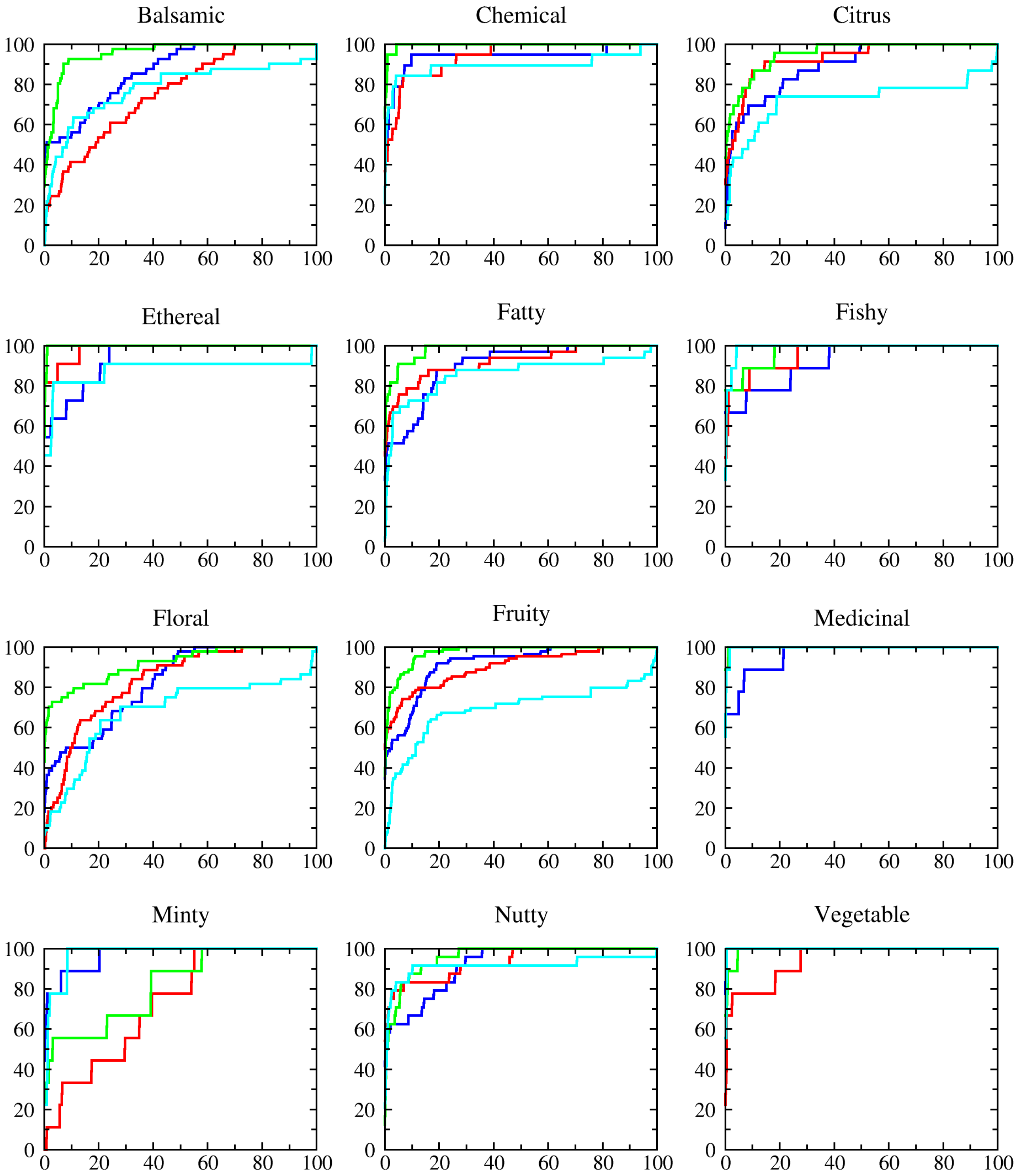

Figure S5. Receiver Operating Characteristic (ROC) curves for recovery of various fragrance classes against GDB-13.FL**,** using CBD_{MQN} (blue), CBD_{Sfp} (red), CBD_{ECfp4} (green), $CBD_{MolWt.}$(cyan). X-axis is % of sorted database and Y-axis is % of fragrances found.
